# A visual analytics approach for understanding biclustering results from microarray data

**DOI:** 10.1186/1471-2105-9-247

**Published:** 2008-05-27

**Authors:** Rodrigo Santamaría, Roberto Therón, Luis Quintales

**Affiliations:** 1 Departamento de Informática y Automática, Universidad de Salamanca, Pz. de Los Caídos S/N, 37007 Salamanca, Spain

## Abstract

**Background:**

Microarray analysis is an important area of bioinformatics. In the last few years, biclustering has become one of the most popular methods for classifying data from microarrays. Although biclustering can be used in any kind of classification problem, nowadays it is mostly used for microarray data classification. A large number of biclustering algorithms have been developed over the years, however little effort has been devoted to the representation of the results.

**Results:**

We present an interactive framework that helps to infer differences or similarities between biclustering results, to unravel trends and to highlight robust groupings of genes and conditions. These linked representations of biclusters can complement biological analysis and reduce the time spent by specialists on interpreting the results. Within the framework, besides other standard representations, a visualization technique is presented which is based on a force-directed graph where biclusters are represented as flexible overlapped groups of genes and conditions. This microarray analysis framework (BicOverlapper), is available at

**Conclusion:**

The main visualization technique, tested with different biclustering results on a real dataset, allows researchers to extract interesting features of the biclustering results, especially the highlighting of overlapping zones that usually represent robust groups of genes and/or conditions. The visual analytics methodology will permit biology experts to study biclustering results without inspecting an overwhelming number of biclusters individually.

## Background

### Biclustering

Microarray experiments determine the transcript abundance of an organism's genes under different conditions. Microarray analysis tries to identify groups of genes that exhibit similar behavior under certain conditions.

One of the main methods to analyze microarray data is biclustering, a non-supervised technique very widespread in the recent years (see [1] for a survey). Biclustering outperforms traditional clustering because of its two main characteristics: simultaneous grouping of genes and conditions, and overlapping. Simultaneous grouping means that biclusters (the groups found by biclustering algorithms) group genes with similar behavior under a certain number of conditions (thus, the bicluster will group genes and conditions), while traditional clustering techniques only group genes with similar behavior across all the conditions (or vice versa). This characteristic makes biclusters better fitted to biological behavior in several circumstances, for example, when an interesting cellular process is active only in a subset of the conditions. Although it is unusual that the subsets of genes grouped by two different clusters intersect, overlapping is an intrinsic characteristic of biclusters. If two biclusters *B*_1 _and *B*_2 _that group genes *G*_1 _and *G*_2 _and conditions *C*_1 _and *C*_2_, respectively, have *G*_1 _∩ *G*_2_≠ ∅ and/or *C*_1 _∩ *C*_2_≠ ∅ it is said that *B*_1 _and *B*_2 _overlap. Overlapping gives biclusters the flexibility to represent biological circumstances such as genes that participate in multiple pathways active under a subset of conditions.

### Visualization of single biclusters

**Figure 1:**
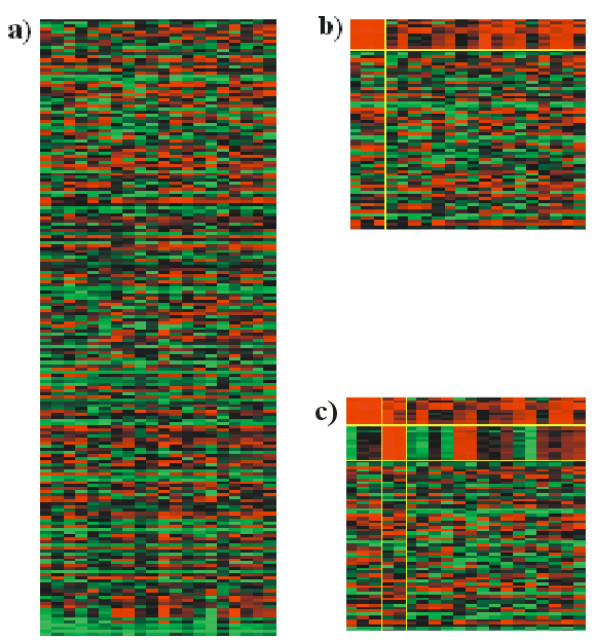
**Heatmap visualization of biclusters**. **a) **Typical heatmap, with bright colors representing the lowest or highest transcription levels. **b) **A section of the heatmap in a), after reordering the rows and columns in such a way as to put those corresponding to an identified bicluster first. **c) **Two non-overlapped biclusters can be seen on the diagonal of the matrix. More non-overlapped biclusters could be added, but if they overlap, representation becomes difficult, or even geometrically impossible without replication of rows and/or columns.

The most widespread visualization technique to represent a single bicluster are heatmaps, which are used in several popular tools [2-4]. In a heatmap (Fig. 1a) genes are displayed as the rows, and conditions as the columns, of a matrix *A*, where element *a*_*ij *_is the transcript abundance of gene *i *under condition *j*. Each element *a*_*ij *_is then represented as a square colored upon its transcript abundance. To draw a bicluster *B*_*k *_that groups a subset of genes *G*_*k *_and conditions *C*_*k*_, the heatmap is reordered so *G*_*k *_rows and *C*_*k *_columns appear together, usually in the upper left section of the matrix (Fig. 1b).

Heatmaps usually satisfy the purpose of inspecting a single bicluster. Unfortunately, they have geometrical limitations when representing several biclusters simultaneously, especially if they overlap (see Fig. 1c). BiVoc [5] addresses this problem by repeating rows and columns to properly represent overlapped biclusters. Although it is a useful tool and implements a method that minimizes the number of repeated rows and columns, this replication could lead to ambiguities and misinterpretations.

**Figure 2:**
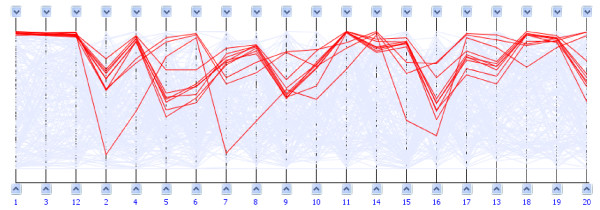
**Parallel coordinates visualization of biclusters**. Parallel coordinates representing the bicluster in Fig. 1b). The lines for genes on the three first conditions are overlapped, indicating a high level of similarity for the genes, equivalent to the homogeneous upper-left area formed in the heatmap.

Parallel coordinates [6] have also been used to represent biclusters, but they are less widespread than heatmaps. In this technique, the gene *g*_*i *_is an *m*-dimensional point *p*_*i *_= (*a*_*i*1_, *a*_*i*2_, ...*a*_*im*_) where *a*_*ik *_is the transcript abundance of *g*_*i *_under condition *c*_*k*_. Conditions are visualized as vertical axes and genes as lines joining the corresponding transcript abundances (Fig. 2).

In this case, a bicluster with *n*' genes and *m*' conditions is represented by *n*' lines corresponding to the genes, and by rearranging or somehow highlighting the axes corresponding to the *m*' conditions. When we try to represent several biclusters with this method, again geometrical problems arise because of the large number of lines and the overlapping of several different ones. BicAT [3] and BiVisu [4] use parallel coordinates to display single biclusters. However, their representations are limited. BicAT does not rearrange bicluster conditions, as it simply marks their corresponding axes with vertical lines (making hard to visualize the whole bicluster). On the other hand, BiVisu only visualizes gene profiles under the conditions in the bicluster, losing context information for other conditions, which could be related but not grouped by the bicluster. None of these methods provide interactive thresholds to manipulate the display.

### Visualization of multiple clusters

**Figure 3:**
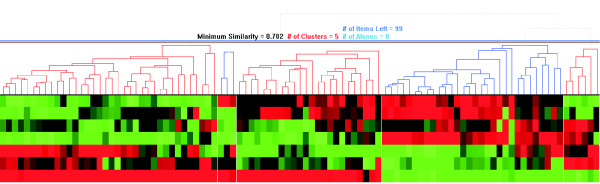
**Dendrogram visualization of hierarchical clustering**. Heatmap and dendrogram representing hierarchical clustering on genes for a yeast microarray data matrix of sporulation conditions, generated with HCE [2]. In this case, a similarity threshold of 0.702 groups genes in five clusters (alternate red and blue branches of the dendrogram), whose differences are clearly visible on the heatmap.

As in the case of single biclusters, the most widespread technique used to visualize multiple clusters from a single clustering are heatmaps. Usually heatmaps are used together with dendrograms, as introduced by Treeview [7]. This way, the hierarchical clustering is represented in a tree and the heatmap rows are rearranged to fit with the clusters found. Sometimes the attached dendrogram can also be used to visually vary the clustering threshold to check the robustness of clusters (see Fig. 3). Usually, clustering is applied to rows (genes) and to columns (conditions), so both dimensions are rearranged and two dendrograms are displayed. Treeview has been enhanced [8], adding a scatterplot visualization for one-by-one condition comparison of transcription levels and a "karyoscope" visualization that represents the transcription levels of the genes under one condition, ordered as they are located in the chromosomes.

**Figure 4:**
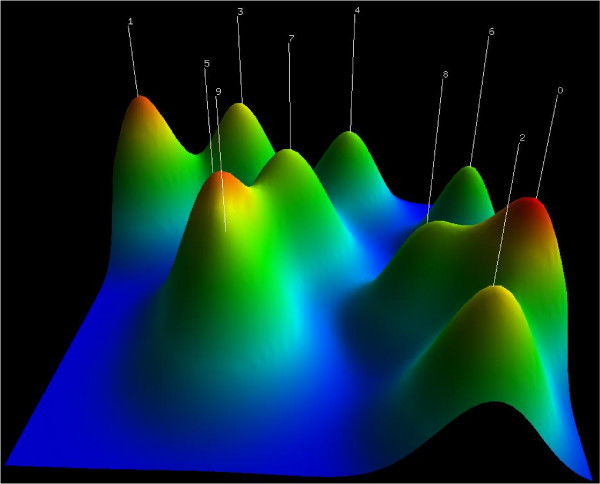
**Mountain map visualization**. gCLUTO's mountain visualization. Each mountain represents a cluster, where position, height, width and slope depend on cluster characteristics such as the genes grouped, the number of genes and their homogeneity.

gCLUTO [9] uses a variation of this heatmap visualization to represent clusters from hierarchical clustering, including the representation of clustering averages for rows and/or columns. In addition, it introduces mountain maps, a 3D visualization technique (see Fig. 4) that displays clusters simultaneously by means of projections onto a 2D space, while the third dimension is used to represent geometrical properties of the mountains (height, width, slope, etc.) that are used to represent properties of the clusters (size, homogeneity, etc.). However, clusters only group genes (not conditions) with similar transcription levels under *all *the conditions, and therefore its adaptation to biclusters is not satisfactory.

Hibbs et al. [10] take advantage of a linked-views approach, so two visualizations, heatmaps and cluster projections, are displayed simultaneously, boosting the visual analysis. The projection used is similar to that of gCLUTO but now in a 3D space. It improves heatmap representation by assigning colors by rank and by visualizing cluster averages. As in gCLUTO, projections are useful, but because of the reduction of dimensionality that they require, some information is lost. Although this is not so important when representing clusters, it becomes an issue with biclusters, where overlapping is a main characteristic and projections usually fail to convey actual overlap between biclusters.

### Related Biological Knowledge

Besides the input (the microarray data matrix) and the outcome of the analysis (for our purpose, biclusters), additional information is available from previous biological studies. This information is usually structured in ontologies, for example, in the case of genes and proteins from eukaryotes, there is the Gene Ontology [11], a dynamic, controlled vocabulary that describes all known biological processes, molecular functions and cellular components associated to them. On the other hand, Transcription Regulation Networks (TRN) represent transcription relationships between genes. In these networks, nodes correspond to genes, and an edge from node *a *to *b *means that gene a transcriptionally regulates (activates or inhibits) gene *b*.

This information can be used to partially guide the bicluster search, or to validate the biclusters found. Note that although this information may be helpful for finding or validating groups, it is rarely complete and grows everyday with new biological discoveries (as an example, the TRN for *Escherichia coli *increased from 577 relationships in 2002 [12] to 2724 in 2004 [13]). Also, its use may bias the search of biclusters to already known groups, limiting the knowledge discovery capability of the methods.

Some of the visualization tools discussed above make use of ontologies to complement their displays, either embedded in the visualization [2] or from a web navigator [8]. There are also several tools that visualize TRNs (for example, Cytoscape [14] or Hawkeye [15]) and link them with other biological knowledge, but it's more difficult to find tools that link TRN networks with clustering or biclustering results.

### Motivation

As described above, the display of several clusters and single biclusters is well known, but the visualization of several biclusters is an almost entirely new area of study. The ability of visualizing several biclusters in the same display speeds up the understanding of relationships among the different biological groups represented by biclusters, specifically it permits:

• To find genes with similar biological functions or conditions that affect similarly to a particular group of genes. This is given by each bicluster alone, but the relevance of these relationships grows if several biclusters coincide in them (forming sorts of 'super-biclusters').

• To trace third-party relationships among biclusters, helping to find, for example, two groups of genes related under different groups of conditions, but also with some conditions in common. The finding of these common genes or conditions ('hubs') is key to infer relationships or bridges among different functional groups.

• To quickly characterize the biclustering algorithm search through its results: is it exhaustive?, does it find several groups?, of which size?, how much are they connected?, are there unconnected groups?

Currently, during an analysis biclusters must be individually inspected and/or filtered using statistical methods or a priori biological knowledge. Due to the heterogeneity of biclustering approaches and the novelty of most of the biclustering algorithms (an increasing number of which have appeared since the year 2000), few theoretical statistical methods to analyze or filter them are available. Most of them are based on significance tests over biological knowledge as Transcription Regulatory Networks [16] or Gene Ontology [17]. These tests are not perfect since biological knowledge is still incomplete. Because of this lack of statistical or biological filters, it is usually difficult to reduce the number of biclusters and even if reduced, to be able to draw conclusions quickly, one way of putting all the biclusters together on a single graphical representation is an urgent need.

Since there are no fault-free standards to determine which is the best biclustering method for each case, problems are usually approached from different points of view, often by using different methods, or different configurations of the same method, in order to identify the most robust results (the biclusters that are found under different approaches).

Due to overlap, in the case of the outcome of a single biclustering method with a single configuration, an interesting fact is that a kind of robustness can still be found in genes or conditions that are grouped together by several biclusters (in other words, they are at the intersection of several biclusters). This can be extended to the use of different methods or configurations of parameters. The robust groups of genes and/or conditions formed by the intersection of different biclusters are a kind of super-biclusters, usually not directly grouped by any method (what can lead to groups of only genes or only conditions) but grouped together by several biclusters.

Visual analytics is the science of analytical reasoning supported by highly interactive visual interfaces [18]. This is our approach, which focuses on the representation of biclusters in several ways that enhance the analysis of biclustering results. Thus, while the center of the analysis is based on a representation of biclusters that is capable of visualizing several biclusters simultaneously, this visualization technique has been implemented as part of a framework that includes other traditional bicluster representations such as heatmaps or parallel coordinates, so the user can inspect biclustering results from different points of view. All the visualizations are highly interactive and are linked together. As a result, the detection of super-biclusters and hub nodes is easy and useful. The framework helps in the comprehension of the differences and similarities among biclusters from different biclustering methods and quickens the task of analyzing biclustering results.

## Results and Discussion

### Group data: movie relations analogy

**Figure 5:**
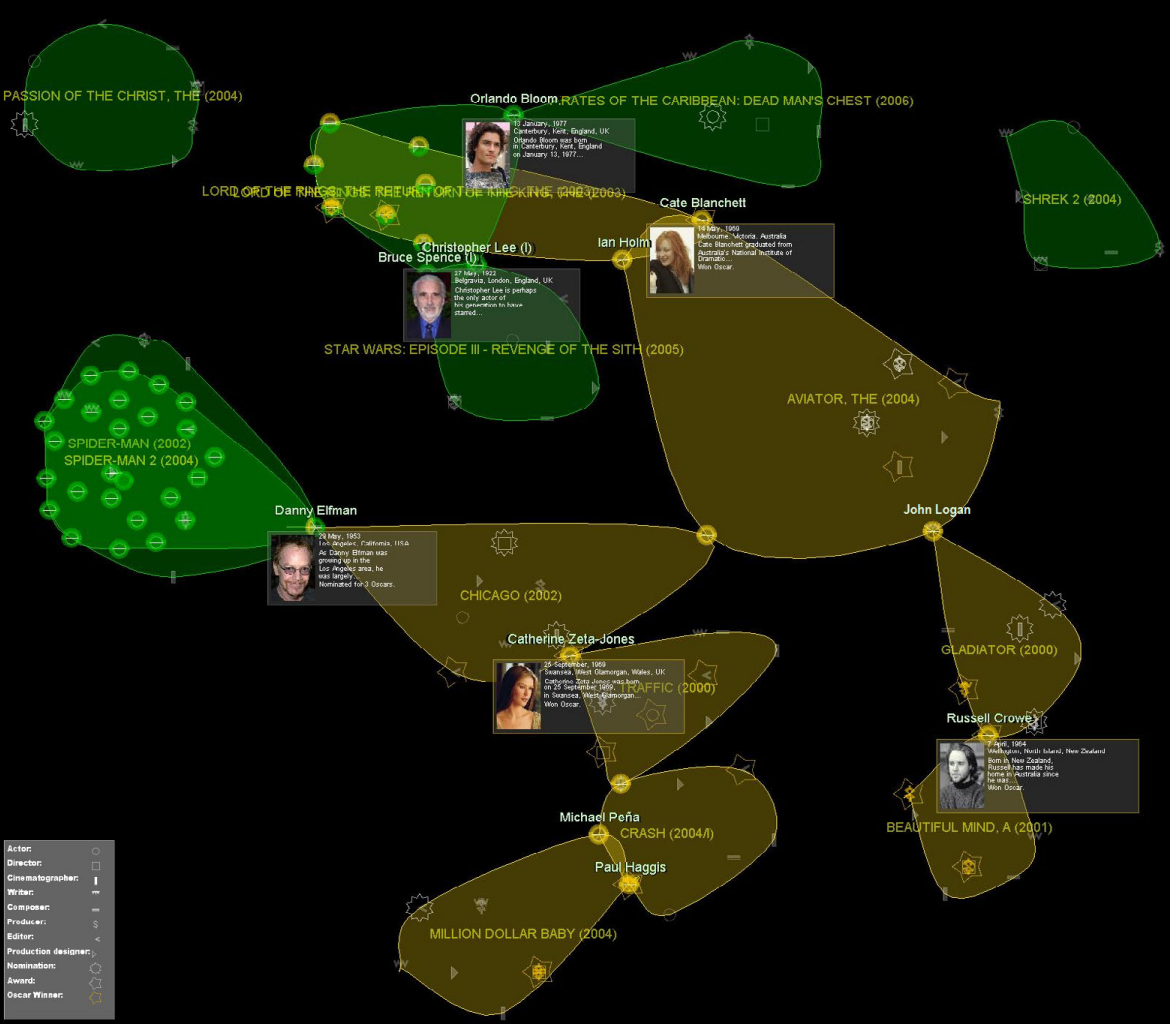
**Movie results visualization**. Groups of people working in the most awarded movies (orange) and the main blockbusters (green) from 2000 to 2006. Recurrent groups appear in blockbusters, referring to the *Spider-Man *saga (left) and the *Lord of the Rings *(top), which also has won several awards. The couple formed by *Michael Peña *and *Paul Haggis *appears in two awarded movies (bottom). Overlapping groups, hub nodes and third party relationships can be quickly identified with this visualization.

The main difficulty when it comes to assessing a visualization for biclustering results is the need for a very well known data set that permits the validation of the conclusions reached using the tool. Taking this into account, before using our visualization technique to represent biclustering results from microarray data sets, it has been validated using a database of more than 20.000 movies and over 250.000 persons, extracted from IMDb [19]. Each movie is treated as a 'bicluster', so each person involved in the movie is a node in it. Of course these are fictional biclusters because they do not come from a biclustering algorithm, nor do they refer to two dimensions, but they have the most interesting property of overlapping. The characteristics that our tool helps to discover in the movie relationships usually have a direct analogy in a biological context, for example:

• Working groups involved in more than one movie. These groups are of special interest if the movies in which the people worked together are prize-winning movies or movies that earned lots of money, because one can identify which are the most successful collaborations. For example, in Fig. 5 both successful sagas (*Spider-man *at the left and *The Lord of the Rings *at the top) and couples working in prize-winning films (such as *Paul Haggis *and *Michael Peña *at the bottom) are easily distinguished. Analogously, groups of genes present in several similar biclusters that are expected to have similar behaviors can be identified, for instance.

• Hub nodes (or groups of nodes) joining two larger, otherwise separated, groups. In the case of movie relationships, these groups are quite interesting because they connect working groups of different nationalities, movie genres or degrees of success. For example, if you are a producer, the hub nodes that join blockbusters with prize-winning movies will lead you to the people that is capable of making quality movies that earn money (in Fig. 5, we can see hub nodes connecting prize-winning movies with blockbusters, such as *Danny Elfman*, and others such as *Catherine Zeta-Jones *or *Orlando Bloom*). In biology hub genes related to two groups of biclusters, each one grouping different biological processes, can be interesting as they may participate in the regulation of both processes.

• Indirect relationships. Each single group gives information of direct relationships among movie people. However, the inspection of several groups, by means of the navigation through the graph (possibly tracing hub nodes), helps to discover third party relationships (notice how, in Fig. 5, *Russell Crowe *and *Cate Blanchett *have worked with *John Logan *in different films). Biologically, this can lead to the discovery of side-effects of the activation (or inhibition) of genes.

The familiarity with the movie ontology makes it easy to test the capability of analysis of the presented technique, much more than to use gene or biological ontologies (usually incomplete), applied to results of (very heterogeneous in concepts) biclustering algorithms. Focused in this field, the framework in which the visualization technique is embedded was also validated by entering two contests, one centered on visual analytics and the other one on graph drawing (social networks). Our entry was selected as finalist of the former [20] and was awarded the first place in the latter [21].

### Microarray Data and Biclustering algorithms

To test the power of our bicluster visualization method, now applied to biological information, the budding yeast *Saccharomyces cerevisiae *microarray data [7] has been used. This data set has been broadly studied and images of heatmap clustering are available. This organism genome is fully sequenced, and the conditions of the microarray are understandable even by non-specialists, presenting clear groups such as sporulation time series, cell division or changes in temperature.

The yeast microarray data forms a 2467 × 79 matrix that has been analyzed using three different biclustering methods: Bimax [16], Iterative Search Algorithm (ISA) [22,23] and Ben-Dor et al. [24] approach to find Order-Preserving SubMatrix biclusters (we will refer to this biclustering algorithm just as OPSM) using BicAT analysis Toolbox [3].

These three algorithms have been chosen because they look for different concepts of biclusters using different strategies. Bimax searches for constant up-regulated biclusters (using Madeira and Oliveira notation [1]), ISA searches for biclusters that highly deviate from the mean (both above or below) and OPSM searches for biclusters which preserve certain order (coherent evolution). Bimax uses a divide-and-conquer strategy while ISA uses Z-score statistics and OPSM performs a greedy iterative search. This way, we can present the results of the visualization under different biclustering conditions and discuss how those differences affect results by comparing their different layouts.

### Bimax results analysis

Bimax is an exhaustive divide-and-conquer method that preprocesses the data matrix to convert it into a binary matrix by fixing a threshold, so transcription levels above this threshold become ones and transcription levels below become zeros (or vice versa). Then, it searches for *all *possible biclusters that contain only ones, so up or down-regulated constant biclusters are found.

Bimax was executed with a discretisation threshold of 1%, so only that percentage of transcription levels (the highest up-regulated) were considered. The minimum size of biclusters was set to 3 × 2, finding 421 biclusters, most of them of small size (groupings with under 30 transcription levels).

**Figure 6:**
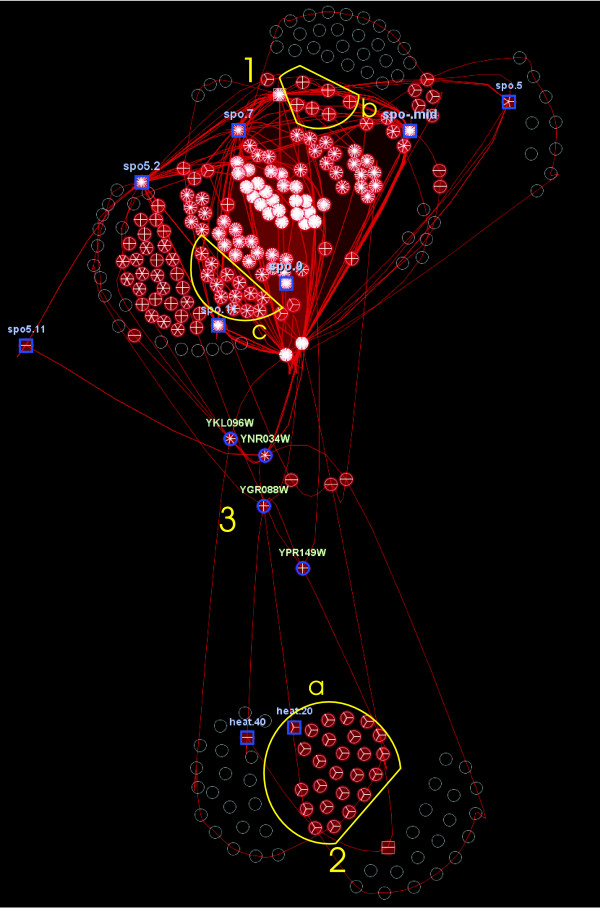
**Bimax results visualization**. Representation of the 50 biggest Bimax biclusters. Clearly, two groups appear at the top (1) and bottom (2), and a small number of genes connecting both groups (3). Subgroups of nodes (mainly genes) grouped together in three (a), four (b) or more (c) biclusters are also easily detected thanks to the pie charts. The names of some relevant genes and conditions have been highlighted.

Fig. 6 shows the 50 biggest biclusters found. With a simple glance at the representation, two clear groups of biclusters appear, one at the top of the graph and another one at the bottom. The display of a higher number of biclusters, up to 250, did not reveal additional information, other than making the two groups tighter.

High connectivity of the nodes demonstrates the exhaustiveness of Bimax, since some of the biclusters are very similar, although no one is completely included in another bicluster. The top group mainly contains conditions related to sporulation (*spo.mid, spo.7, spo.9, spo.11 *are the most biclustered but not the only ones), revealing that this process provokes up-regulation on a high number of genes. We have compared Fig. 6 with the genes related to sporulation that have been identified by Eisen et al. [7] by means of clustering. The top group contains all the genes related to sporulation in that previous work, as expected. The bottom group, less connected, contains biclusters that group other conditions, especially heat shock conditions such as *heat.20 *and *heat.40*. Some genes highly active under heat shock and sporulation conditions such as those with ORFs *YGR088W*, *YNR034W *or *YKL096W *are present in biclusters of both groups; and can be seen at the center of the representation. These hub genes are of special biological interest because they act as a bridge between sporulation conditions and heat shock conditions. For example, ORF *YKL096W *corresponds to gene *CWP1*, involved in cell wall organization [25] and known to be related to sporulation [26], but it has not been related with heat shock conditions, triggering a new research question in order to clarify these findings.

### OPSM results analysis

OPSM defines a bicluster as a group of rows whose values are monotonically increased under a certain column ordering, enabling us to find coherent evolution biclusters, i.e. genes and conditions that significatively increase or decrease at the same time regardless of the amount of the change. This is the broadest bicluster definition, yielding sometimes very large groups of genes.

OPSM was run using 10 models for each iteration, which yielded 13 biclusters. Four of the biclusters found were ignored due to their high number of genes (above 400).

**Figure 7:**
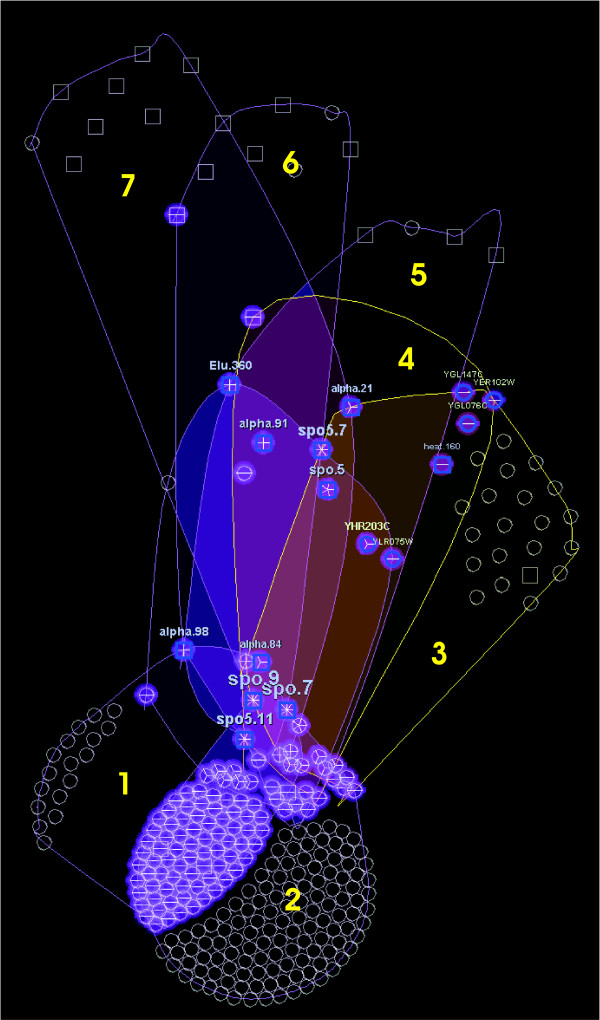
**OPSM results visualization**. OPSM bicluster visualization. Biclusters 3 and 4 are highlighted after interaction with the representation. Biclusters grouping mainly genes (1, 2, 3) or mainly conditions (6, 7) are easily identified. The relaxed condition of coherent evolution makes biclusters very large in some cases (1, 2).

The visualization (Fig. 7) reveals one of the characteristics of OPSM biclusters: when an OPSM bicluster contains few genes, it usually has more conditions, and vice versa (this is especially evident in biclusters 6 and 7, or 1 and 2). However the most interesting result that the visualization helps to quickly detect for this dataset is that OPSM biclusters are mainly connected by sporulation conditions. These detected conditions are biologically interesting because they are able to maintain an order in transcription levels over a large number of genes.

This feature could also be discovered by means of the visualization of single biclusters, but it requires much more effort. Also, third party relationships cannot be discovered unless all the elements in each bicluster are tracked one by one, while in this visualization they are quickly identified. For example, we can see that genes with locus tags *YGL147C*, *YER102W *and *YGL076C *are grouped together in two biclusters (3 and 4), and are not related to genes in bicluster 1, except for some nodes (mainly sporulation conditions) at the center. These three genes, along with several others, such as *YHR203C *or *YLR075W *(highlighted in the figure), are protein components of the ribosomal subunits *40S *and *60S*. This explains why they are grouped together in biclusters by OPSM. In this case, they serve as validation of the method because there are biological evidence of the relation among genes (components of ribosomal subunits), but in other cases (as for example, in Bimax hub nodes above) these identifications could lead to new knowledge. It is also remarkable that most of the genes grouped along with sporulation conditions at OPSM is not grouped by Bimax for the same conditions, suggesting that genes related to ribosomal subunits present order in transcription levels during sporulation, but they are not highly expressed.

### ISA results analysis

Iterative Search Algorithm (ISA) aims at finding genes and conditions that deviate from the mean, so only highly up- or down-regulated genes and conditions are biclustered. The method starts with two normalized copies of the data matrix, one for genes and another one for conditions. Then, different thresholds are imposed for genes and conditions, and biclusters are searched using Z-score statistics. In the end, biclusters with both up- and down-regulated transcription levels are obtained.

**Figure 8:**
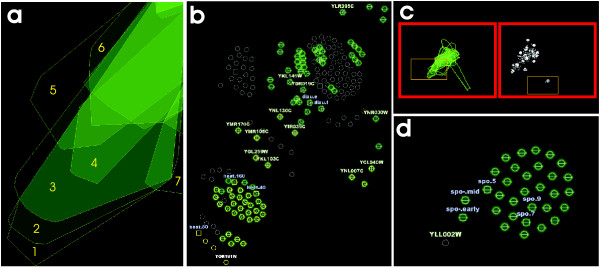
**ISA results visualization**. Detail of ISA biclusters. **a) **Hulls wrapping highly overlapped biclusters. Biclusters 1, 2 and 3 share nodes, but elongated hulls tell us they also share other nodes with biclusters 4, 5, and 6. Bicluster 7 also shares some nodes with biclusters 1, 2 and 3. **b) **The same biclusters as in a), but hulls are hidden and glyphs drawn. This detailed representation complements and clarifies hull representation, focusing on overlapping groups of genes and conditions. All nodes related to *YOR181W *at the bottom-that is, all nodes grouped by bicluster 1- are highlighted in bright yellow. **c) **Overviews of the complete set of ISA biclusters, with and without hulls. The orange rectangles in the overview are used to select the desired area of detail (left: overview of a) and b), right: overview of d)). **d) **Detail of sporulation-related biclusters. In this case, only two biclusters support this relationship, much less important for ISA than for Bimax or OPSM.

This algorithm found 45 biclusters with both gene and condition thresholds set to 2, and taking 100 starting points. ISA's bicluster structure is more entangled than the ones of Bimax or OPSM (see Fig. 8). While hull overlapping helps to draw conclusions regarding bicluster relationships (see Fig. 8a), when clusters grow in number and heterogeneity as in this case, abstraction to a higher level of grouping is also interesting. This way, highly intersected zones, such as nodes in biclusters 1, 2 and 3 (Fig. 8b) acquire relevance not through the individual biclusters they pertain to, but through the frequency by which the biclustering method groups them together (forming a super-bicluster around conditions *heat.40 *and *heat.160*). When complexity increases, it is also interesting to know exactly what nodes are connected, which is achieved by highlighting all related nodes when hovering one of them.

Since ISA searches for both up and down-regulated biclusters, relevant nodes differ from Bimax. For example, some conditions arise as important for this method, such as heat shock conditions *heat.40*, *heat.80*, *heat.160 *or diauxic shift conditions *diau.e*, *diau.f *(see Fig. 8b), while sporulation conditions, very relevant in Bimax, are secondary (Fig. 8d).

## Conclusion

The present article analyzes and compares results from three prominent biclustering methods when applied to a real microarray experiment using a visual analytics framework that allows whole representation and interaction for all biclusters. The main conclusions are the following:

• The proposed visualization allows to display large number of biclusters in a single representation, enhancing the detection of overlap among biclusters.

• As a consequence of conveying overlapping groups, actual biological features can be extracted by the detection of super-biclusters and hub nodes.

• The combination of different representations (hulls, piecharts, labels) with the interaction and navigation through the graph helps in the analysis, allowing to simplify the visualization of complex results.

• This visualization also helps to determine biclustering algorithms characteristics, and differences and similarities between biclustering algorithms.

• The integration of the presented visualization into a visual framework that provides standard representations helps experts to follow the results more easily. Furthermore, the linkage of novel and traditional visualizations permits a deeper analysis of results, from overview to details, thus gaining insight into the problem at hand.

Following these promising results, our future line of work will be based on the research and optimization of the layout when different biclustering algorithm's results are compared with each other, and on the integration of additional biological knowledge from gene and condition databases.

## Methods

**Figure 9:**
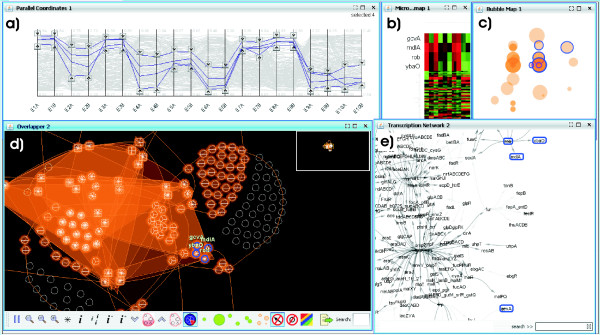
**Framework**. The framework, showing all the implemented visualization techniques: **a) **Parallel coordinates. **b) **Microarray heatmap. **c) **Bicluster bubble map. **d) **Overlapper. **e) **TRN graph. The figure shows data from a synthetic *Escherichia coli *microarray, generated by SynTReN [40], and the biclusters found on it by Turner's plaid model [34]. In the figure, four gene nodes have been selected in the overlapper (d). These genes are grouped together in several biclusters, forming a super-bicluster. Its selection has flown to other views, modifying them. This way it can be easily determined that they actually have similar gene profiles (a, b). Also, we can see that all of them are in the same transcription group, except one, *gcvA *(e).

This section details the main characteristics of the presented visual analytics approach, focusing on the description of the novel graph-based bicluster visualization (we will refer to it as *overlapper*) and its use inside a framework (*BicOverlapper*) that implements other well known bicluster visualizations such as heatmaps or parallel coordinates (see Fig. 9).

We start with the definition of bicluster, then we explain the graph building, layout and complexity. Finally, we will see how the overlapper and the other views interact and are linked together to help discover new knowledge.

### Bicluster Definition

**Figure 10:**
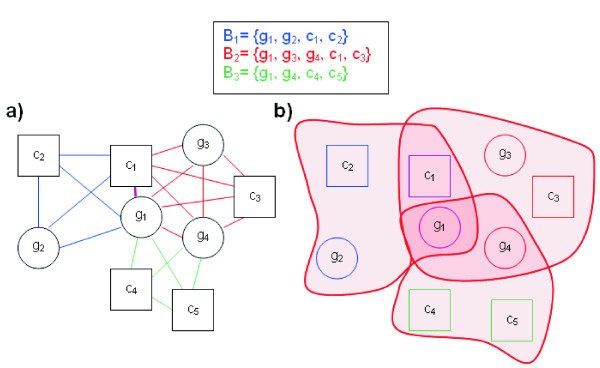
**Bicluster building**. This figure shows how three simple biclusters (*B*_1_, *B*_2_, *B*_3_) are represented. Observe that *g*_1 _is in the three biclusters, and that *c*_1 _and *g*_4 _are in two of them. **a) **The method generates an internal edge structure by building complete graphs for each subset of nodes corresponding to a bicluster. This structure places close nodes in the same biclusters when the force layout is applied, moving *g*_1 _to the center of the three biclusters, since it is shared by all of them. A similar behavior occurs with *c*_1 _and *g*_4_, shared each one by two different biclusters. **b) **When displayed, edges are omitted and instead hulls with limits defined by splines surrounding the outermost nodes of each bicluster are drawn. Hulls are transparent, so their overlap forms solider colors, highlighting *g*_1 _more than *c*_1 _and *g*_4_, and these ones more than the rest.

The presented visualization technique relies on a graph where nodes represent genes or conditions, and edges join nodes that are grouped by one or more biclusters (Fig. 10). By using the same entity (graph nodes) for genes and conditions, the characteristic of the grouping of genes and conditions, natural in biclusters, can be easily visualized, a difficult task when both entities are separated (rows and columns in heatmaps, or lines and axes in parallel coordinates). Of course, gene nodes and condition nodes will be finally identifiable by using different shapes to represent them.

Let *B*_*k *_be a bicluster that groups genes *G*_*k *_= {*g*_*k*1_, ..., *g*_*kn*_} and conditions *C*_*k *_= {*c*_*k*1_, ..., *c*_*km*_}. *B*_*k *_is represented as an undirected complete subgraph with nodes *N*_*k *_= {*G*_*k *_∪ *C*_*k*_} = {*g*_*k*1_, ..., *g*_*kn*_, *c*_*k*1_..., *c*_*km*_}. As previously explained, for purposes of graph computation, genes and conditions are not distinguished and are simply considered as nodes *N*_*k *_= {*n*_1_, ..., *n*_*k*(*n*+*m*)_}, and edges are defined as *E*_*k *_= {(*n*_*i*_, *n*_*j*_) = (*n*_*j*_, *n*_*i*_), *with n*_*i*_, *n*_*j *_∈ *N*_*k*_}. The weight *w*_*ij *_of each edge *e*_*ij *_= (*n*_*i*_, *n*_*j*_) is given by the number of biclusters that contain both nodes *n*_*i *_and *n*_*j*_.

### Graph Layout

The nodes are displayed following a force-directed layout [27]. In our model, each pair of nodes *n*_*i *_and *n*_*j *_(positioned at **p**_**i **_and **p**_**j **_respectively) with a distance between them **d**_**ij**_, can be affected by up to two forces. If the nodes are connected, a spring force acts to keep them at an optimal distance *d*_*o*_, with stiffness depending on a constant s and the weight of the edge:

(1)

where |**d**_**ij**_| = |**p**_**j **_- **p**_**i**_| is the magnitude of the distance and ${\hat{d}}_{ij}$ is the unit vector that indicates direction from *n*_*i *_to *n*_*j*_. Between every pair of nodes, whether connected or not, an expansion force makes them repel each other:

(2) $$X_{ij}=-(G/|d_{ij}|){\hat{d}}_{ij}$$

where *G *is a gravitational constant that controls the intensity of the repulsion. **S**_**ij **_keeps nodes in the same biclusters close, while **X**_**ij **_separates nodes into different biclusters.

Edge cluttering is an issue when we deal with large graphs, making the resulting display confusing [28]. To solve this, edges are not drawn unless requested by the user. Instead, each bicluster (represented as a complete subgraph) is wrapped in a polygon or a rounded shape (hull). This hull is drawn by determining the outermost nodes of each bicluster, and using their positions as anchor points for a spline curve that draws the contour of the hull. The inside of the hull is filled with the same color used for the line, but with a degree of transparency. Unlike other zone graph visualizations [29,30], a node can be in more than one zone, reflecting overlapping between biclusters, which can usually affect more than one node. Because hulls are drawn with a transparent color, their intersecting areas become more opaque, enhancing the detection of overlaps.

### Node Representation

**Figure 11:**
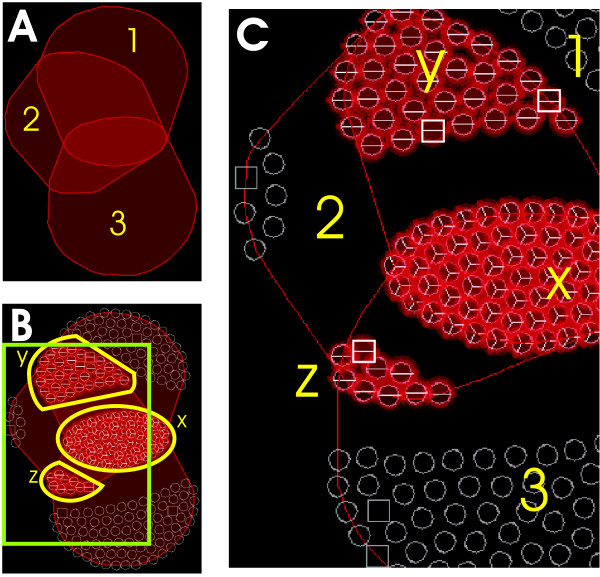
**Bicluster visualization**. Three of the biggest biclusters that Bimax algorithm found for microarray data discussed in the Results section. **a) **Biclusters represented by their wrapping hulls. This visualization is useful to draw an overview of how biclusters are related. **b) **Nodes are now represented as glyphs. Attention goes into intersecting areas, which can be seen as 'supergroups' of genes and/or conditions (x, y, z). These structures will frequently appear in more complex visualizations. **c) **Detail of b). The nodes in a supergroup are drawn as pie charts. These nodes have a strong relationship under the specific criteria of the biclustering algorithm. For example, in the case of (z), it means that eight genes have very high transcription levels with similar variations under two subsets of conditions (those from biclusters 2 and 3). These two subsets share one condition (square shaped), present in the supergroup. In addition, we can see that biclusters 1 and 2 share two conditions; also there are no conditions shared by the three biclusters; finally bicluster 2 is secondary, because it only has six genes and one condition not grouped in biclusters 1 or 3.

Node positions are defined by the graph layout, but other information can be displayed by node representation, by means of glyphs, at user's demand. A glyph is a graphical object designed to convey multiple data values [31]. The geometrical properties of the glyph represent different dimensions (Fig. 11). In our case:

• The shape of each node distinguishes between genes (circles) and conditions (squares).

• Pie charts have as many sectors as biclusters in which the node appears. The color of sectors could also be used to identify different biclusters which meet some predetermined criterion. Pie charts also serve to quantify the degree of overlapping of hull zones.

• Labels with gene and condition names can be displayed for node identification. In this case, label color is determined by the node type (gene or condition) and text size by the degree of overlapping.

The final result of the graph display is a set of flexible overlapped, colored areas representing biclusters, with glyph nodes inside representing genes or conditions. Drawing these areas instead of drawing edges, along with its flexibility, allows a large number of biclusters to be represented without excessive cluttering on the display.

### Graph Complexity

An optimal implementation of force-directed layouts has a complexity of *O*(*n*^3^) [32], with *n *being the number of nodes. Microarray experiments tend to have high dimensionality, containing 10^3–5^genes and 10^1–2 ^conditions. Usually the number of genes *n *is much higher than the number of conditions *m*.

Regarding edge complexity on our representation, the worst scenario for an *n *× *m *microarray data matrix is that all genes behave similarly for all conditions, so the only bicluster will be the entire matrix *n *× *m*, and thus the resulting graph will have *n *+ *m *nodes and (*n *+ *m*)(*n *+ *m *- 1)/2 edges (around 10^6 ^edges for a 1000 × 100 matrix).

Obviously, such a microarray experiment is useless, but helps to understand that complexity is very sensitive to the dimensionality of the biclusters. The number of biclusters found is another factor which increases the complexity, but is much less important, since more edges will be shared when the number of biclusters grows.

In practice, the number and size of biclusters vary depending on the biclustering algorithm, the input parameters and the microarray data set. Typically, for a 1000 × 100 matrix, an exhaustive method like Bimax [16] gives hundreds of biclusters. Other methods such as Spectral Biclustering [33] or ISA [22] yield around 50 biclusters, while Turner's Plaid model [34] or OPSM [24] generate only a dozen biclusters. Other algorithms take the number of biclusters as an input parameter. Usual sizes for biclusters range from 2 × 2 to 100 × 10, though exceptionally larger ones may be generated.

Overlapper can deal with up to 100 biclusters with sizes ranging from 2 × 2 to 100 × 10, on an Intel Pentium D 2.8 GHz processor, without relevant loss of interactivity. Although performance is currently being optimized to deal with more than 100 biclusters, graph complexity and the ability of human perception to inspect a graph impose limits on the number of biclusters than can be visualized. Therefore, previous statistical or biological filters linked to graph visualization are of great importance when it comes to comparing larger biclustering results.

The visual analytics approach described here helps to reduce complexity by interacting with other linked visualizations such as parallel coordinates or TRN networks, filtering the displayed biclusters using simple criteria such as "only biclusters that contains gene X" or "only biclusters with genes that have high transcription levels for condition Y".

### Multiple Linked Views

Four other visualizations are implemented along with the overlapper: heatmap, parallel coordinates, TRN network and bubble map. Heatmap and parallel coordinates are used to display transcription levels and also to represent gene or condition profiles and single biclusters.

**Figure 12:**
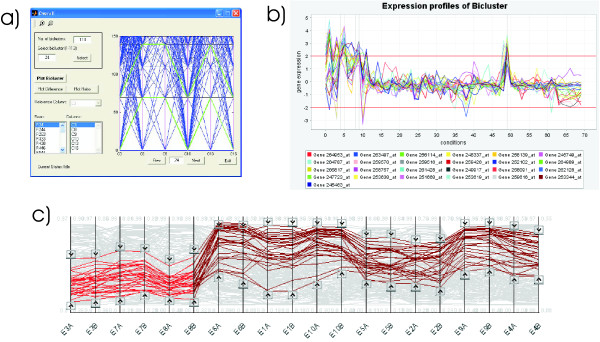
**Parallel coordinates comparison**. **a) **BiVisu parallel coordinates. All gene expression profiles are displayed, with the ones in the bicluster in green and the rest in blue, but only conditions in the bicluster are displayed. The highest and lowest expression levels are hard to tell. **b) **BicAT parallel coordinates. Gene profiles of the genes in the bicluster are displayed through all the conditions. The conditions in the bicluster are marked by a vertical line, but they are not rearranged, so visual interpretation of the bicluster is difficult. Gene profiles not in the bicluster are not drawn, and no interaction is implemented. **c) **BicOverlapper parallel coordinates. Gene profiles in the bicluster are highlighted in red. The rest of gene profiles are still visible as a light gray background. Coordinate axes corresponding to conditions in the bicluster are rearranged on the left, and gene profile segments for conditions in the bicluster are highlighted in bright red. Interaction with the parallel coordinates, allowing threshold setting and rearrangement of axes is implemented.

The heatmap implementation is conventional, representing single biclusters by rearranging and distorting the corresponding rows and columns. The implementation of parallel coordinates allows transcription thresholds to be set for each condition, thus helping to perform user-driven filtering of gene profiles. Biclusters are represented by first placing all coordinate axes corresponding to conditions in the bicluster. The lines corresponding to genes in the bicluster are highlighted, with the segments corresponding to conditions in the bicluster being brighter (see Fig. 12). With this implementation of parallel coordinates, the context of genes and conditions not in the bicluster is not lost, one of the main limitations of other implementations of this technique, as discussed in the Background section.

On the other side, the bubble map is a 2D projection map similar to gCLUTO's mountain map, but we use both genes and conditions to compute projections (see Fig. 9c). However, this implementation uses 2D characteristics such as diameter, color and transparency to represent the characteristics of biclusters (size, homogeneity, etc.), so biclusters are drawn as 2D 'bubbles' instead of 3D mountains. This way, we avoid the occlusion of objects, which is an issue in 3D visualizations, and we simplify the display, which is more complex in the case of biclusters than in the case of clusters.

Finally, the TRN visualization is implemented as a force directed graph in the fashion of tools such as Cytoscape [14] or Hawkeye [15], with genes as nodes, joined by directed edges if they are related by any transcription behavior (activation or inhibition).

Visual analytics focuses on the interaction with the representations, so they can be adapted to the user's information needs. Thus, all the visualizations in the framework, including the overlapper, implement a large number of options to interact with them. Most of these interactions deal with navigation through the view and the capability of selecting or searching for biclusters, genes or conditions. However, other interactions are specific to each visualization, such as for example, the setting of transcription thresholds in parallel coordinates, as described above. Please refer to the Implementation section for a list of additional material explaining these interactions in detail.

It must be added that many of the interactions within a representation will lead to different visual changes in the rest of the visualizations of the framework.

### Filters and thresholds

A helpful way of using the proposed visual analytics methodology is an incremental exploration of the problem. The initial problem (the analysis of all the biclusters found for a given microarray data matrix) can be divided into simpler problems. For example, if a gene is considered interesting for our experiment, to search for all the biclusters that group it can be a way to simplify our analysis.

For this reason, as stated above, all the visualizations implemented in the framework are linked, so an interaction with one of them propagates to the other visualizations. By interacting with the ancillary visualizations (heatmap, parallel coordinates, TRN network and bubble map) we can filter the number of biclusters displayed in the overlapper making them easier to analyze.

**Figure 13:**
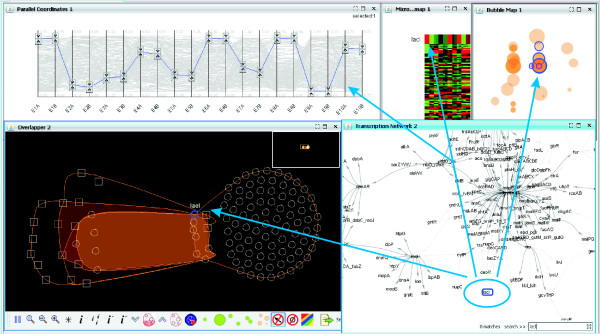
**Gene lacI represented in the framework**. The gene *lacI *has been selected in TRN network (the example corresponds to the same data in Fig. 9). The selection propagates to other views, highlighting the corresponding profile in the heatmap and parallel coordinates. The overlapper display is greatly simplified with respect to Fig. 9, and reveals that *lacI *is grouped with six other genes and two conditions in five biclusters. This group of eight nodes recurrently grouped together may be worth further study if *lacI *is important for the analyst.

**Figure 14:**
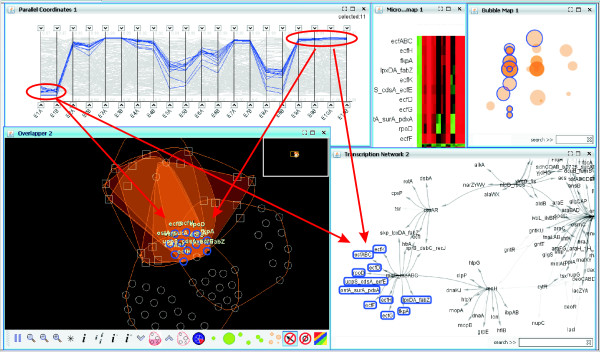
**Selection of transcription levels in the framework**. In the same example as Fig. 13, now parallel coordinates are used to select the genes with high transcription levels in the conditions *E9A, E9B, E10A *and *E10B *and also low transcription levels for conditions *E1A, E1B*. The result are eleven genes that form the core of several biclusters. All these genes have a high correlation under all the conditions, as shown by the parallel coordinates and the heatmap. If we analyze the TRN network, we see that this occurs because all of them correspond to the same activation branch of the network.

For example, we can search for a gene named *lacI *in the TRN network and select it to display its profile (parallel coordinates, heatmap) and the biclusters where it belongs to, which will appear in the bubble map and the overlapper (see Fig. 13). Or we can set parallel coordinate thresholds to select the genes with low transcription levels for conditions *E1A*, *E1B *and high levels for conditions *E9A*, *E9B*, *E10A *and *E10B*, and see which biclusters contain them all and how they are related within the TRN network (see Fig. 14). In addition to this multiple-view filtering, the overlapper visualization alone allows the setting of internal thresholds that also help to simplify the visualization and to focus on bicluster subsets. Three kind of thresholds are available to modify the display:

• *Overlap threshold*: when this node-oriented threshold is set to *n*, only genes and conditions grouped in more than *n *biclusters are drawn.

• *Size threshold*: when this bicluster-oriented threshold is set to *n*, only biclusters with at least *n *nodes (counting genes and conditions) are drawn.

• *Constance threshold*: if this threshold is set to *n*, only biclusters with standard deviation less than *n *are drawn. Note that some biclustering algorithms do not use constance as a criterion to find biclusters, so this threshold will disfavor them.

Both filters and thresholds are useful to focus on specific subgroups of genes or conditions, but without them the overlapper is still capable of visualizing several biclusters and allowing the user to draw conclusions about the behavior of biclustering algorithms and the biological data grouped by them, as is discussed in the Result section.

### Implementation

The visual analysis approach described has been implemented as a Java framework called BicOverlapper. The overlapper technique was initially designed as a sketch in Processing [35], and later was translated to pure Java [36]. Heatmap, TRN network and bubble map implementations make use of the Prefuse library [37].

A public version of BicOverlapper is available, and can be executed in any operating system that supports Java 1.6. The framework makes use of three different sources of data:

• The bicluster results, which contain all the biclusters to be visualized in the overlapper.

• The microarray data matrix, necessary for the visualization of heatmaps and parallel coordinates.

• The TRN network with information about transcription regulations and necessary for the TRN visualization.

**Figure 15:**
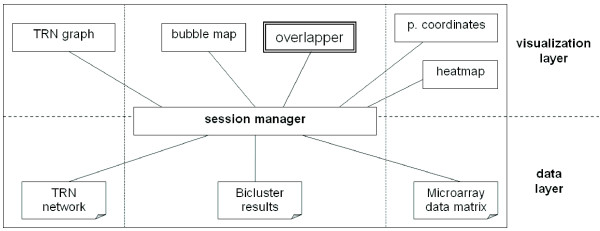
**Framework architecture**. Diagram for the structure of the framework. Three data sources can be used in the visualization of different displays, by means of a session manager that interconnects them all.

Although these data sources are fundamentally different, they all share genes and conditions as elementary entities, so the different visualizations in the framework can be linked by them. Basically, this is done by a *session manager *that separates the different data sources from the visualizations, filtering the relevant data entities (see Fig. 15). When a change happens in one of the visualizations because of the user interaction, the session manager detects and translates it to the associated changes in the rest of linked visualizations. More technical information about the framework, their options and implementation details can be found in the following documents:

• A user's guide with installation instructions, further information about usage of the software, interaction options and a complete case study.

• A developer's guide explaining details about the architecture and design of the Java implementation.

• A technical report with a review of the state of the art and details on the visualization technique and the framework.

All these files, along with the open source and a ready to use distribution of the framework are available at the BicOverlapper development site [38]. The framework and the user's guide are also available for download [39].

## Authors' contributions

RT and RS conceived the study and carried out the computational analysis, implementation and optimization. RT and LQ managed and coordinated the project. LQ studied biological data mining issues and RT and RS studied visual analysis issues. All authors participated in writing and revising the final manuscript.

## Supplementary Material

Additional file 1 Shockwave Flash (swf) video showing overlapper interaction. Click here for file

Additional file 2 Shockwave Flash (swf) video showing Bimax biclusters in overlapper. Click here for file

Additional file 3 Shockwave Flash (swf) video showing OPSM biclusters in overlapper. Click here for file

## References

[B1] Madeira S, Oliveira A (2004). Biclustering algorithms for biological data analysis: a survey. IEEE/ACM Trans Comput Biol Bioinform.

[B2] Seo J, Shneiderman B (2002). Interactively Exploring Hierarchical Clustering Results by Interactive Exploration of Dendrograms, a Case Study with Genomic Microarray Data. IEEE Computer.

[B3] Barkow S, Bleuer S, Prelic A, Zimmermann P, Zitzler E (2006). BicAT: a biclustering analysis toolbox. Bioinformatics.

[B4] Cheng KO, Law NF, Siu WC, Lau TH (2007). BiVisu: Software Tool for Bicluster Detection and Visualization. Bioinformatics.

[B5] Grothaus GA, Mufti A, Murali T (2006). Automatic layout and visualization of biclusters. Algorithms for Molecular Biology.

[B6] Inselberg A (1985). The plane with parallel coordinates. The Visual Computer.

[B7] Eisen MB, Spellman PT, Brown PO, Botstein D (1998). Cluster analysis and display of genome-wide expression patterns. Proc Natl Acad Sci USA.

[B8] Saldanha AJ (2004). Java Treeview-extensible visualization of microarray data. Bioinformatics.

[B9] Rasmussen M, Karypis G (2004). gCLUTO: An Interactive Clustering, Visualization and Analysis System. Tech Rep 04–021.

[B10] Hibbs MA, Dirksen NC, Li K, Troyanskaya OG (2005). Visualization methods for statistical analysis of microarray clusters. BMC Bioinformatics.

[B11] Ashburner M, Ball CA, Blake JA, Bolsteing D, Butler H, Cherry JM, Davis AP, Dolinski K, Dwight SS, Eppig JT, Harris MA, Hill DP, Issel-Tarver L, Kasarskis A, Lewis S, Matese JC, Richardson JE, Ringwald M, Rubin GM, Sherlock G (2000). Gene Ontology: tool for the unification of biology. The Gene Ontology Consortium. Nature Genetics.

[B12] Shen-Orr SS, Milo R, Mangan S, Alon U (2002). Network motifs in the transcriptional regulation network of Escherichia coli. Nature Genetics.

[B13] Ma HW, Kumar B, Ditges U, Gunzer F, Buer J, Zeng AP (2004). An extended transcriptional regulatory network of Escherichia coli and analysis of its hierarchical structure and network motifs. Nucleic Acids Research.

[B14] Shannon P, Markiel A, Ozier O, Baliga NS, Wang JT, Ramage D, Amin N, Schwikowski B, Ideker T (2003). A Software Environment for Integrated Models of Biomolecular Interaction Networks. Genome Res.

[B15] Schatz MC, Phillippy AM, Shneiderman B, Salzberg SL (2007). Hawkeye: an interactive visual analytics tool for genome assemblies. Genome Biology.

[B16] Prelic A, Bleuer S, Zimmermann P, Wille A, Buhlmann P, Gruissem W, Hennig L, Thiele L, Zitzler E (2006). A systematic comparison and evaluation of biclustering methods for gene expression data. Bioinformatics.

[B17] Carmona-Saez P, Pascual-Marqui RD, Tirado F, Carazo JM, Pascual-Montano A (2006). Biclustering of gene expression data by non-smooth non-negative matrix factorization. BMC Bioinformatics.

[B18] National Visualization and Analytics Center (2005). Illuminating the Path: The Research and Development Agenda for Visual Analytics.

[B19] Internet Movie Database. http://www.imdb.com.

[B20] Theron R, Santamaria R, Garcia J, Gomez D, Paz-Madrid V (2007). Overlapper: movie analyzer. Information Visualization Confererence Compendium.

[B21] Duncan CA, Kobourov SG, Sander G (2007). Graph Drawing Contest Report. Lecture Notes in Computer Science, GD'07.

[B22] Ihmels J, Friedlander G, Bergmann S, Sarig O, Ziv Y, Barkai N (2002). Revealing modular organization in the yeast transcriptional network. Nat Genet.

[B23] Ihmels J, Bergmann S, Barkai N (2004). Defining transcription modules using large-scale gene expression data. Bioinformatics.

[B24] Ben-Dor A, Chor B, Karp R, Yakhini Z (2003). Discovering local structure in gene expression data: the order-preserving submatrix problem. Journal of Computational Biology.

[B25] Vaart JM van der, Caro LH, Chapman JW, Klis FM, Verrips CT (1995). Identification of three mannoproteins in the cell wall of Saccharomyces cerevisiae. Journal of Bacteriology.

[B26] Tevzadze GG, Swift H, Esposito RE (2000). Spo1, a phospholipase B homolog, is required for spindle pole body duplication during meiosis in Saccharomyces cerevisiae. Chromosoma.

[B27] Fruchterman TMJ, Reinhold EM (1991). Graph Drawing by Force-directed Placement. Software – Practice and Experience.

[B28] Gansner ER, North SC (1998). Improved force-directed layouts. Proc of the 6th Symposium on Graph Drawing.

[B29] Perer A, Shneiderman B (2006). Balancing Systematic and Flexible Exploration of Social Networks. IEEE Trans on Vis and Comp Graphics.

[B30] Kumar G, Garland M (2006). Visual Exploration of Complex Time-Varying Graphs. IEEE Trans on Vis and Comp Graph.

[B31] Ware C (1999). Perception for Design.

[B32] Herman, Melançon G, Marshall MS (2000). Graph Visualization and Navigation in Information Visualization: A Survey. IEEE Trans on Vis and Comp Graph.

[B33] Kluger Y, Basri R, Chang JT, Gerstein M (2003). Spectral Biclustering of Microarray Data: Coclustering Genes and Conditions. Genome Res.

[B34] Turner HL, Bailey TC, Krzanowski WJ, Hemingway CA (2005). Biclustering Models for Structured Microarray Data. IEEE/ACM Trans Comput Biol Bioinform.

[B35] Fry B, Reas C (2007). Processing, a programming handbook for visual designers and artists.

[B36] Santamaría R, Therón R, Quintales L (2008). BicOverlapper: A tool for bicluster visualization. Bioinformatics.

[B37] Heer J, Card SK, Landay JA (2005). prefuse: a toolkit for interactive information visualization. Proceedings of SIGCHI Human Factors in Computing Systems.

[B38] Bicoverlapper Development Site. http://bicoverlapper.googlecode.com.

[B39] BicOverlapper. http://vis.usal.es/bicoverlapper.

[B40] den Bulcke TV, Leemput KV, Naudts B, van Remortel P, Ma H, Verschoren A, Moor BD, Marchal K (2006). SynTReN: a generator of synthetic gene expression data for design and analysis of structure learning algorithms. BMC Bioinformatics.

